# Lack of Effect of Cenerimod, a Selective S1P_1_ Receptor Modulator, on the Pharmacokinetics of a Combined Oral Contraceptive

**DOI:** 10.3390/ijms232314986

**Published:** 2022-11-29

**Authors:** Pierre-Eric Juif, Markus S. Mueller, Hakim Charfi, Jasper Dingemanse

**Affiliations:** 1 Department of Clinical Pharmacology, Idorsia Pharmaceuticals Ltd., Hegenheimermattweg 91, 4123 Allschwil, Switzerland; 2 Biotrial, 7–9 Rue Jean-Louis Bertrand, 35000 Rennes, France

**Keywords:** cenerimod, pharmacokinetics, sphingosine-1-phosphate, levonorgestrel, ethinylestradiol

## Abstract

Cenerimod, a sphingosine-1-phosphate 1 receptor modulator, is in development for the treatment of systemic lupus erythematosus, a disease mainly affecting women of childbearing potential. The effect of cenerimod on the pharmacokinetics (PK) of a combined oral contraceptive (COC, 100 µg levonorgestrel and 20 µg ethinylestradiol (EE)) was investigated. A randomized, double-blind, parallel-group study was performed in 24 healthy male and female subjects. A single oral dose of COC was administered alone and after 35 days of once daily (o.d.) administration of cenerimod 0.5 (n = 10) or 4 (n = 14) mg. Exposure to EE alone or in combination with cenerimod was comparable as reflected by the geometric mean ratios and the respective 90% confidence intervals, while a slight increase in exposure (approximately 10–25%) to levonorgestrel was observed at clinically relevant concentrations of cenerimod. Overall, COC alone or in combination with cenerimod was safe and well tolerated. Two subjects reported one adverse event each (one headache after COC alone, and gastroenteritis in combination with cenerimod 4 mg). In conclusion, cenerimod does not affect the PK of levonorgestrel or EE to a clinically relevant extent. Therefore, COC can be selected as method of contraception during and after cenerimod therapy without the risk of interaction.

## 1. Introduction

Administration of sphingosine-1-phosphate 1 receptor (S1P_1_R) modulators triggers a sustained internalization of this receptor and induces a long-lasting inhibition of the egress of lymphocytes from lymphoid organs, suggesting efficacy in autoimmune disorders [1].

Cenerimod is an orally available, potent, and selective modulator of the S1P_1_R under clinical development as potential treatment for systemic lupus erythematosus (SLE) [2,3,4]. The pharmacokinetics (PK) of cenerimod have been extensively characterized in healthy subjects and revealed a time (t_max_) to maximum plasma concentration (C_max_) of 5–6 h, terminal half-life (t_½_) of 12–22 days after multiple-dose administration, and dose-proportional exposure [3]. Steady-state conditions were observed after approximately 4 weeks of once daily (o.d.) dosing [3]. The absorption and elimination of cenerimod are slower compared to other S1P_1_R modulators [5,6,7]. Cenerimod displays a cytochrome P450 (CYP) enzyme-independent metabolism, and no major metabolites were found in plasma. It is primarily excreted in feces with a single major metabolite formed CYP-independently by reductive cleavage [8]. Its PK and pharmacodynamics are not affected by ethnicity in healthy Asian and white subjects [5]. In vitro data showed that cenerimod is glucuronidated in the liver by UDP-glucuronosyltransferase (UGT) 2B7 and 1A6, is a substrate of a single transporter (breast cancer resistance protein), and is not a perpetrator of any CYP- or transporter-mediated drug-drug interaction (DDI).

SLE is an autoimmune disease that causes multiorgan inflammation, directly impacts patients’ quality-of-life, and can be severely disabling. It affects more women than men, with a sex ratio of between 2:1 and 15:1 [9]. Symptoms and diagnosis of SLE generally occur between the age of 15 and 44 years, thereby predominantly affecting women of childbearing potential. As observed with other S1P_1_R modulators, cenerimod has shown teratogenic potential in preclinical toxicology. Therefore, to allow for the administration of cenerimod in women of childbearing potential, they must not be pregnant and must use a highly effective contraceptive method. Although in vitro experiments indicate no flag for DDI with hormonal contraceptives, there is a need to investigate the potential reduction in the efficacy of hormonal contraception when contraceptive steroids are given on top of the investigational medicinal product [10]. This study was conducted to investigate the effect of cenerimod at doses of 0.5 and 4 mg at steady state on the PK of a combined oral contraceptive (COC), i.e., 100 µg levonorgestrel and 20 µg ethinylestradiol (EE).

## 2. Results

### 2.1. Disposition and Demographics

The demographics were well balanced across the two treatment groups, and the overall mean (range) age and body mass index (BMI) were 45.8 (24–65) years and 23.6 (20.0–29.8) kg/m^2^, respectively. Demographics are presented in Table 1.

### 2.2. PK

**Figure 1:**
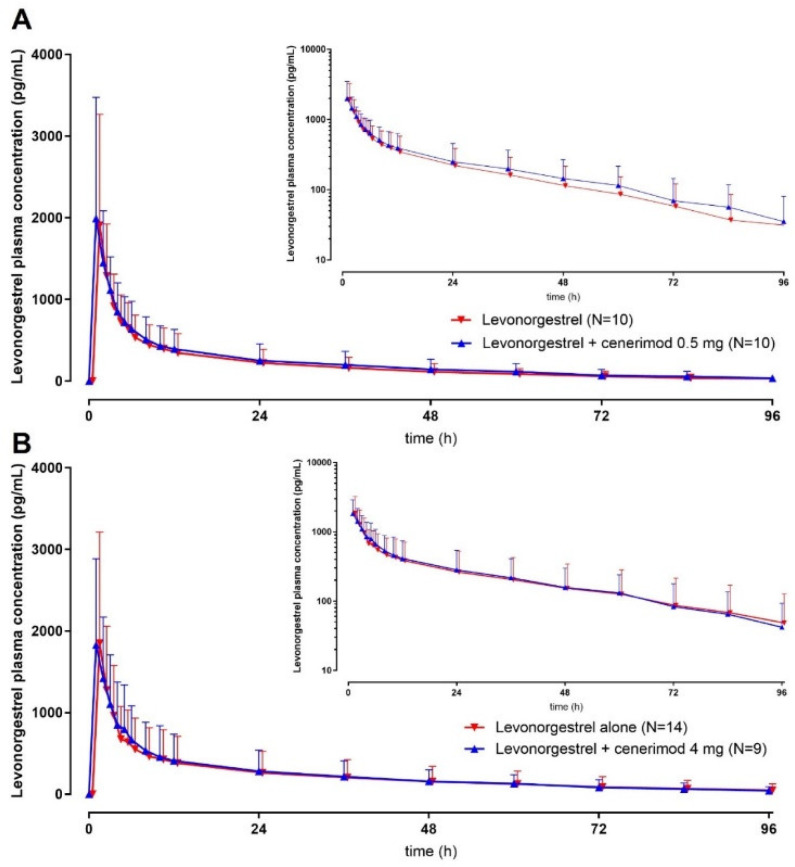
Plasma concentration profile of levonorgestrel vs. time over the duration of the study in the absence and presence of cenerimod 0.5 (Panel **A**) or 4 (Panel **B**) mg. In total, 24 subjects were enrolled in this study (10 in COC + cenerimod 0.5 mg, 14 in COC + cenerimod 4 mg), and 5 subjects discontinued study treatment prior to the second administration of COC (all in the COC + cenerimod 4 mg group).

**Figure 2:**
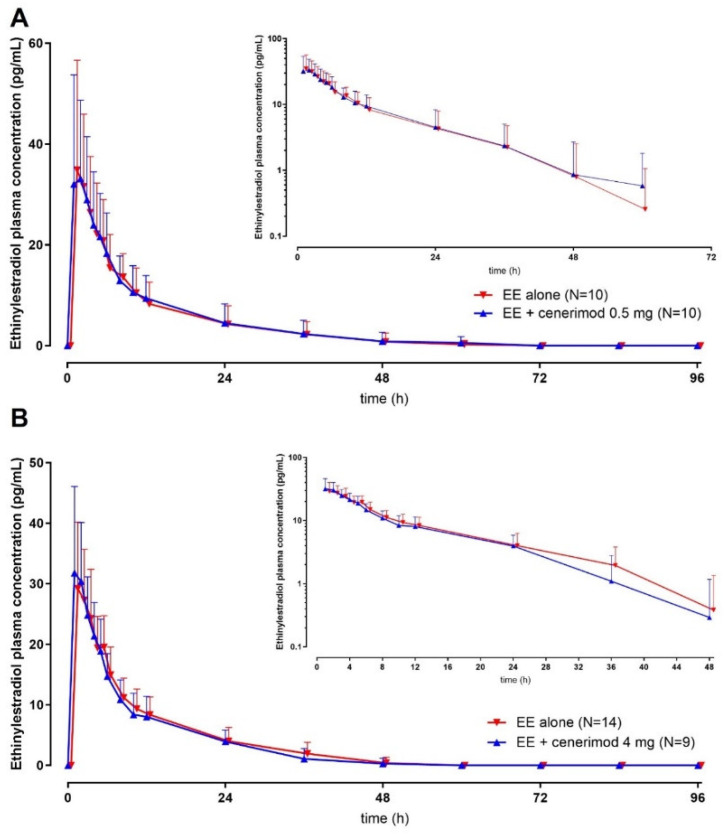
Plasma concentration profile of ethinylestradiol (EE) vs. time over the duration of the study in the absence and presence of cenerimod 0.5 (Panel **A**) or 4 (Panel **B**) mg. In total, 24 subjects were enrolled in this study (10 in COC + cenerimod 0.5 mg, 14 in COC + cenerimod 4 mg), and 5 subjects discontinued study treatment prior to the second administration of COC (all in the COC + cenerimod 4 mg group).

Plasma concentration vs. time profiles of levonorgestrel and EE are depicted in Figure 1 and Figure 2, respectively, and the PK parameters are presented in Table 2 and Table 3, respectively. No differences in PK were apparent between males and females; therefore, subjects from both sexes were combined.

### 2.3. Effect of Cenerimod on the PK of Levonorgestrel

At clinically relevant concentrations of cenerimod, exposure to levonorgestrel was slightly increased as reflected by the GMR of C_max_ and the area under the plasma concentration-time curve (AUC) >1 (Table 2). The 90% confidence intervals (CI) included 1 for C_max_ (0.98–1.26 for cenerimod 0.5 mg and 0.93–1.21 for cenerimod 4 mg) but not for AUC from 0 to the last measurable time point (AUC_0–t_) and AUC from 0 to infinity (AUC_0–∞_, Table 2). The median t_max_ was similar in the absence and presence of cenerimod.

The GMR values were also around or slightly above 1 for t_½_. The 90% CI included 1 for cenerimod 0.5 mg (0.92–1.09) but not for cenerimod 4 mg (1.02–1.23). 

### 2.4. Effect of Cenerimod on the PK of EE

At clinically relevant concentrations of cenerimod, exposure to EE was very similar to the exposure assessed when given alone as shown by GMR values around 1 and the 90% CI including 1 for C_max_, AUC_0–t_, and AUC_0–∞_. The t_max_ and t_½_ of EE were also not affected by cenerimod, as presented in Table 3.

### 2.5. Safety and Tolerability

In this study, no serious adverse events were reported. Two subjects reported one adverse event (AE) each. One subject reported one AE of headache after COC alone and one subject reported an AE of gastroenteritis after COC + cenerimod 4 mg. Only the AE of headache was assessed by the investigator as related to study treatment, and both AEs were of moderate intensity.

Out of the 24 subjects who participated in the present study, 5 subjects prematurely discontinued from study treatment, i.e., 4 subjects due to AEs of lymphopenia (a total lymphocyte count between 0.2 and 0.5 × 10^9^ cells/L) and 1 subject due to an AE of ECG T-wave inversion. However, as prospectively planned in the statistical analysis plan, these AEs were not assigned to the periods COC alone or COC + cenerimod as they occurred after the period in which COC was administered alone and before the period in which COC was to be administered on top of cenerimod.

During the COC periods, there were no clinically relevant ECG abnormalities. Other safety parameters were also comparable between treatments (i.e., COC alone or COC + cenerimod 0.5 or 4 mg).

Taken together, the administration of COC alone or in combination with cenerimod was safe and well tolerated.

## 3. Discussion

Oral contraceptives are the most used method of contraception, with 17% of women aged 15–49 years using oral contraceptives in the US [11]. As this method is one of the most preferred in preventing pregnancy, a significant need exists to understand the DDI potential of investigational drugs. This is of particular relevance for teratogenic products so that evidence-based guidance on reliable contraceptive methods can be provided to women taking these drugs [12]. In this respect, the Clinical Trial Facilitation Group released a guidance highlighting the need for a DDI study with contraceptive steroids despite the lack of DDI potential in vitro of the investigational drug [10]. 

The PK and metabolism of levonorgestrel and EE have been extensively investigated. Both levonorgestrel and EE are eliminated primarily by hepatic metabolism. CYPs are the major pathways in the metabolism of levonorgestrel (CYP3A4 and 3A5) and EE (CYP3A4) [13,14].

The effect of fingolimod [15], siponimod [16], ponesimod [17], and ozanimod [18] on the PK of oral contraceptives has been previously investigated. For fingolimod and siponimod, a combination of levenorgestrel and EE was used, while norethisterone and EE were investigated for ponesimod and ozanimod. None of these S1P_1_R modulators, which have a teratogenic potential, affect the PK of COC to a clinically relevant extent. 

The PK of cenerimod are characterized by a long t_½_ of 12–22 days after multiple-dose administration of 0.5–4 mg and a high accumulation ratio of 7–9 [3]. Due to the slow accumulation of cenerimod, this study was designed to administer COC at clinically relevant steady-state concentrations of cenerimod. 

In the present study, the plasma concentration vs. time profiles as well as the PK parameters of EE and levonorgestrel were in line with published data [15,16].

The PK of EE were not affected by o.d. dosing with cenerimod 0.5 or 4 mg, while the exposure to levonorgestrel was slightly increased by cenerimod. This increase was of approximately 10 and 20–25% as reflected by the GMRs of C_max_ and AUC, respectively (Table 2 and Table 3). The same pattern was observed for fingolimod and siponimod, i.e., the AUC of levonorgestrel (but not EE) increased by 22 and 28%, respectively. The effect on C_max_ was less marked (10% for fingolimod and 18% for siponimod).

One limitation of this study might be that it employed a precision-estimate approach to define the sample size for investigating the effect of cenerimod on the PK of COC. Despite that, the increase in exposure to levonorgestrel is not considered clinically relevant. The same conclusion was drawn for siponimod and fingolimod [15,16]. The lack of a clinically significant effect of cenerimod on the PK of levonorgestrel and EE was expected based on the absence of the perpetrator potential of cenerimod on drug metabolizing enzymes involved in the metabolism of levonorgestrel and EE.

Additionally, it must be considered that the effect observed was an increase in exposure; therefore, there is no reduced efficacy of COC when given on top of clinically relevant concentrations of cenerimod. The incidence and frequency of safety events of interest related to EE (e.g., thromboembolism) are not expected to be affected by the absence of the effect of cenerimod on the PK of COC.

In this study, male and female subjects received COC. To our knowledge, the inclusion of male subjects in DDI studies with COC is uncommon. The inclusion of male subjects and the limited number of female subjects enrolled (women being more affected by SLE than men) may be seen as a limitation of the study. However, this was justified as no pharmacodynamic assessments were planned and subjects were their own control, i.e., the GMRs were calculated for each individual, and then mixed-effect models were applied, limiting the potential sex-related bias. Therefore, data gathered in both male and female subjects are considered reliable to support the absence of a clinically relevant effect of cenerimod on the PK of COC. There is no expectation that without a clinically relevant effect on the exposure to COC, the efficacy of contraception in women with SLE would be affected.

COC alone or in combination with cenerimod was safe and well tolerated in this study with two AEs of moderate intensity reported.

In conclusion, this study demonstrated that cenerimod does not affect the PK of COC to a clinically relevant extent, and the combination was safe and well tolerated, confirming COC as an option for effective contraception in patients receiving cenerimod. 

## 4. Materials and Methods 

### 4.1. Study Design

The study was conducted at a single center in Rennes, France, (Biotrial), in accordance with the Declaration of Helsinki and Good Clinical Practice. All participants provided written informed consent prior to any study-related procedures. The protocol was submitted to the French Health Authorities and an Independent Ethics Committee (CPP Ile de France IV, Paris, France). Both approved the study. 

The present work was a sub-study in a larger thorough QT study (NCT04255277) in which 97 subjects were enrolled. This was a randomized, double-blind-for-cenerimod, open-label-for-COC, parallel-group study evaluating the PK, safety, and tolerability of a single dose of a COC. Subjects received a single dose of COC on Day 1, cenerimod 0.5 or 4 mg o.d. on Days 7–56, and a second dose of COC on Day 42. This design was selected based on the long t_½_ of cenerimod, which precluded a cross-over study design.

Based on a maximum coefficient of variation (CV) of 40.1% [19], with a sample size of 20 evaluable subjects (10 per dose group of cenerimod) and assuming a ratio of 1, it was estimated that the 90% CI for the GMR COC + cenerimod/COC alone would be approximately 0.79, 1.27. 

### 4.2. Study Population

A total of 10 (6 male and 4 female) subjects were randomized in the COC + cenerimod 0.5 mg group and 14 (9 male and 5 female) subjects in the COC + cenerimod 4 mg group. Five (3 male and 2 female) subjects in the COC + cenerimod 4 mg group discontinued study treatment prior to Day 42 (i.e., the day of COC administration on top of cenerimod) due to AEs, which occurred outside the prospectively defined AE reporting period for COC alone or COC + cenerimod. Therefore, 19 subjects (10 in cenerimod 0.5 mg and 9 in cenerimod 4 mg) completed study treatment with COC. 

The healthy status of the subjects was determined based on the absence of any active or chronic disease, complete physical examination, vital signs, 12-lead ECG, and clinical laboratory data. At Screening and on Day −1, systolic/diastolic blood pressure and heart rate had to be in the range of 90–145/45–90 mmHg and 50–100 bpm, respectively.

### 4.3. Study Conduct

Subjects were admitted to the clinic the day before dosing, after a screening period (10–28 days for women of childbearing potential and 2–28 days for other subjects). On Day 1 and Day 42, a single oral dose of COC was administered as a tablet formulation under fasted conditions (i.e., the last food intake at least 12 h prior to dosing). From Day 7 to Day 56 (i.e., 14 days after the second administration of COC), cenerimod 0.5 or 4 mg was administered o.d. in the morning. The selection of the 0.5 and 4 mg doses was based on the doses tested in the dose-finding phase 2 study (NCT03742037).

Each subject remained at the study site until discharge 48 h after dosing with COC followed by ambulatory visits every 12 h for 5 days after COC administration. After the assessment of the PK of COC, subjects remained in the larger TQT study.

### 4.4. Drug Selection

The COC used here was a low-dose combination of EE/levonorgestrel 20 µg/100 µg. Since it combined progestogen and estrogen, it allowed for the investigation of the effect of cenerimod on the PK of both components. The drug administered (Leeloo^®^) was commercially available in France.

### 4.5. PK Assessments

Blood samples of approximately 3 mL for each analyte (i.e., levonorgestrel and EE) were collected in ethylene diamine tetraacetic acid (EDTA) tubes pre-dose and at 1, 2, 3, 4, 5, 6, 8, 10, 12, 24, 36, 48, 60, 72, 84, and 96 h post-dose. After centrifugation, plasma was transferred into a polypropylene tube and stored at −20 (±5) °C until analysis. Plasma concentrations of levonorgestrel and EE were determined using a validated liquid chromatography coupled to tandem mass spectrometry (LC-MS/MS) assay with a lower limit of quantification (LLOQ) of 25 pg/mL for levonorgestrel and 2.5 pg/mL for EE. The assay method involved liquid–liquid extraction, chromatographic separation on a Phenomenex X-Bridge BEH C18 column under gradient conditions with 0.1% formic acid in water and acetonitrile/2-propanol (9/1, *v/v*) as mobile phases, and MS/MS detection using [^2^H_4_]-EE and [^2^H_6_]-levonorgestrel as internal standards. A turbo ion spray was used as the ion source. Measurements were performed in the positive ionisation mode. The LC-MS/MS assay was conducted and validated by Nuvisan GmbH (Neu-Ulm, Germany) in accordance with internationally accepted standards. The acceptance criteria had an overall accuracy ±15.0% from nominal concentration at all levels and precision ≤15.0%. Mass transitions were 530.2 > 171.3, 313.1 > 245.1, 534.2 > 171.3, and 319.1 > 251.1 for EE, levonorgestrel, [^2^H_4_]-EE, and [^2^H_6_]-levonorgestrel, respectively. The method was linear in the concentration range 25–2500 and 2.5–250 pg/mL for levonorgestrel and EE, respectively. An analysis of quality-control samples of all runs showed that inter-batch coefficients of variation (precision) were <6.3 and <14.2% for levonorgestrel and EE, respectively, whereas the average intra-batch accuracy was in the range between −5.3–0.5 and −2.9–5.1% for levonorgestrel and EE, respectively. 

Noncompartmental PK analyses were performed using Professional WinNonlin 8.0 software (Certara, Princeton, NJ, USA). C_max_ and t_max_ were directly obtained from the plasma concentration–time profiles. AUC_0–t_ was calculated using the trapezoidal method [20]. AUC_0–∞_was calculated by combining AUC_0–t_ and AUC_extra_. AUC_extra_ represents an extrapolated value obtained by C_t_/λ_z_, where C_t_ is the last plasma concentration measured above the LOQ and λ_z_ the elimination rate constant determined by log-linear regression analysis. The t_1/2_ was calculated as ln 2/λ_z_.

The effect of cenerimod on the C_max_, AUC_0–t_, AUC_0–∞,_ and t_½_ of COC was investigated using the ratio of the geometric means and 90% CI, with COC treatment alone as the reference treatment and COC + cenerimod as the test treatment. The log-transformed values were analyzed by mixed-effects models including the treatment and dose of cenerimod as fixed effects and the subject as a random effect. The ratios of geometric means and their 90% CIs were calculated from the corresponding back log-transformed contrasts of the mixed-effects models for the C_max_, AUC_0–t_, AUC_0–∞_, and t_½_ of COC alone or COC + cenerimod.

### 4.6. Safety and Tolerability Assessments

Safety and tolerability were evaluated based on AE, vital signs, 12-lead ECG, and clinical laboratory data, as well as physical examinations. Only the AEs reported during the COC period, i.e., for 5 days after administration of COC, are reported here.

## Figures and Tables

**Table 1:** Subject demographics.

	Statistic	Cenerimod 0.5 mg (N = 10)	Cenerimod 4 mg (N = 14)	Overall (N = 24)
**Sex**				
**Female**	**n (%)**	4 (40.0 %)	5 (35.7 %)	9 (37.5 %)
**Male**	**n (%)**	6 (60.0 %)	9 (64.3 %)	15 (62.5 %)
**Age (years)**	**Mean (SD)**	49.5 (15.06)	43.2 (13.93)	45.8 (14.44)
	**Range**	26–65	24–64	24–65
**Weight (kg) ^1^**	**Mean (SD)**	68.46 (12.799)	70.51 (10.190)	69.65 (11.129)
	**Range**	45.8–90.1	50.6–83.8	45.8–90.1
**Height (cm)**	**Mean (SD)**	171.3 (11.53)	171.6 (9.29)	171.5 (10.04)
	**Range**	151–186	154–185	151–186
**BMI (kg/m^2^)**	**Mean (SD)**	23.24 (3.290)	23.93 (2.840)	23.64 (2.986)
	**Range**	20.1–29.8	20.0–28.9	20.0–29.8

% = Percentage of subjects based on N; BMI = body mass index; N = number of subjects in the treatment group; n = number of subjects with available data; and SD = standard deviation. ^1^ Body weight measured at screening.

**Table 2:** Summary of PK variables of levonorgestrel in the absence and presence of cenerimod 0.5 (n = 10) or 4 (n = 9) mg at steady state, and effect of cenerimod on the PK of levonorgestrel.

	PK of Levonorgestrel			Effect of Cenerimod on the PK of Levonorgestrel	
	COC alone	COC + cenerimod 0.5 mg	COC + cenerimod 4 mg	COC + cenerimod 0.5 mg vs. COC alone	COC + cenerimod 4 mg vs. COC alone
	(N = 24)	(n = 10)	(n = 9)		
C_max_ (pg/mL)	1571 (1195–2066)	1778 (1153–2742)	1597 (1038–2456)	1.11 (0.98–1.26)	1.06 (0.93–1.21)
				0.1538	0.4404
					
t_max_ (h)	1.00 (1.00–3.00)	1.00 (1.00–2.00)	1.00 (1.00–2.00)	NC	NC
			
AUC_0–t_ (h·pg/mL)	15,307 (11,129–21,055)	17,876 (11,145–28,673)	17,930 (10,034–32,038)	1.18 (1.11–1.25)	1.22 (1.14–1.30)
				0.0002	<.0001
AUC_0–∞_ (h·pg/mL)	17,395 (12,728–23,772)	20,084 (12,852–31,386)	20,570 (11,831–35,764)	1.17 (1.10–1.25)	1.25 (1.17–1.34)
				0.0004	<.0001
					
t_½_ (h)	27.4 (23. 5–31.9)	28.4 (23.5–34.4)	28.9 (22.6–36.9)	1.00 (0.92–1.09)	1.12 (1.02–1.23)
				0.9655	0.0445

Data are expressed as geometric mean (95% confidence interval) for COC alone, COC + cenerimod 0.5 mg, and COC + cenerimod 4 mg. The effect of cenerimod on the PK of COC is expressed as ratio of geometric mean (90% confidence interval) with *p*-value for COC + cenerimod vs. COC alone. In total, 24 subjects were enrolled in this study (10 in COC + cenerimod 0.5 mg, 14 in COC + cenerimod 4 mg), and 5 subjects discontinued study treatment prior to the second administration of COC (all in the COC + cenerimod 4 mg group). AUC_0−∞_, area under the plasma concentration-time curve from time zero to infinity; AUC_0−t_, area under the plasma concentration-time curve from time zero to time t of the last measured concentration above the limit of quantification; C_max_, maximum plasma concentration; COC, combined oral contraceptive; N, number of subjects in the population; n, number of subjects with available data; NC, not calculated; t_½_, terminal half-life; t_max_, time to reach C_max_; and PK, pharmacokinetics.

**Table 3:** Summary of PK variables of ethinylestradiol in the absence and presence of cenerimod 0.5 (n = 10) or 4 (n = 9) mg at steady state and effect of cenerimod on the PK of ethinylestradiol.

	PK of Ethinylestradiol			Effect of Cenerimod on the PK of Ethinylestradiol	
	COC alone	COC + cenerimod 0.5 mg	COC + cenerimod 4 mg	COC + cenerimod 0.5 mg vs. COC alone	COC + cenerimod 4 mg vs. COC alone
	(N = 24)	(n = 10)	(n = 9)		
					
C_max_ (pg/mL)	31.1 (26.1–37.2)	32.1 (21.7–47.5)	32.1 (24.0–42.9)	0.98 (0.85–1.14)	1.08 (0.92–1.26)
				0.8480	0.4100
t_max_ (h)	1.00 (1.00–3.00)	2.00 (1.00–3.00)	1.00 (1.00–3.00)	NC	NC
			
AUC_0–t_ (h·pg/mL)	266 (205–345)	285 (169–483)	270 (195–374)	1.10 (0.95–1.27)	1.04 (0.90–1.21)
				0.2603	0.6447
AUC_0–∞_ (h·pg/mL)	334 (269–416)	345 (212–563)	325 (237–447)	1.03 (0.92–1.16)	1.00 (0.89–1.13)
				0.6581	0.9798
t_½_ (h)	12.1 (10.1–14.5)	10.7 (6.94–16.4)	10.6 (7.06–15.8)	0.92 (0.81–1.06)	0.88 (0.76–1.01)
				0.3182	0.1324

Data are expressed as geometric mean (95% confidence interval) for COC alone, COC + cenerimod 0.5 mg, and COC + cenerimod 4 mg. The effect of cenerimod on the PK of COC is expressed as ratio of geometric mean (90% confidence interval) with *p*-value for COC + cenerimod vs. COC alone. In total, 24 subjects were enrolled in this study (10 in COC + cenerimod 0.5 mg, 14 in COC + cenerimod 4 mg), and 5 subjects discontinued study treatment prior to the second administration of COC (all in the COC + cenerimod 4 mg group). AUC_0−∞_, area under the plasma concentration-time curve from time zero to infinity; AUC_0−t_, area under the plasma concentration-time curve from time zero to time t of the last measured concentration above the limit of quantification; C_max_, maximum plasma concentration; COC, combined oral contraceptive; N, number of subjects in the population; n, number of subjects with available data; NC, not calculated; t_½_, terminal half-life; t_max_, time to reach C_max_; and PK, pharmacokinetics.
